# Do toddlers’ levels of cortisol and the perceptions of parents and professional caregivers tell the same story about transition from home to childcare? A mixed method study

**DOI:** 10.3389/fpsyg.2023.1165788

**Published:** 2023-06-02

**Authors:** May Britt Drugli, Kathrin Nystad, Stian Lydersen, Anne Synnøve Brenne

**Affiliations:** ^1^Department of Mental Health, Faculty of Medicine and Health Science, Regional Centre for Child and Youth Mental Health and Child Welfare, Norwegian University of Science and Technology (NTNU), Trondheim, Norway; ^2^Centre of the Study of Educational Practice, Inland Norway University of Applied Sciences, Trondheim, Norway

**Keywords:** transition, childcare, toddler, cortisol, mixed method

## Abstract

**Introduction:**

Enrolling in childcare represents the first transition in toddlers’ lives and lays the foundation for their well-being in childcare centers going forward. Child cortisol levels may be an indicator of how toddlers themselves experience their introduction to childcare. In the present study, we explored changes in toddler cortisol levels during their first month in childcare and at a 3-month follow-up, as well as the perceptions of parents and professional caregivers regarding the toddlers’ settling-in process during the same period.

**Method:**

This study used a mixed-method design. Saliva samples were collected from 113 toddlers and their cortisol levels analyzed. Qualitative notes were collected from parents (*n* = 87) and professional caregivers (*n* = 101). The data were analyzed using linear mixed model and thematic analyses, respectively.

**Results:**

Changes in toddler cortisol levels and their parents’ and professional caregivers’ perceptions of the transition process fit well. Both data sources indicated an easy start in childcare when parents were present, while the first weeks separated from parents seemed quite demanding. After 3 months, the cortisol levels returned to a low level, while child well-being was perceived as high.

**Discussion:**

Toddlers need time to adapt to childcare settings. Even if they are well taken care of by their keyworkers during the day, many toddlers are tired and exhausted in the evenings at home, particularly in the first weeks of separation from their parents. Both professional caregivers and parents should be aware of toddlers’ need for emotional support during their transition to childcare.

## 1. Introduction

Today, most children in the Western world attend childcare from an early age. In Norway, 87% of toddlers (children aged 1–2 years) are in childcare centers, and many of them for long hours (Statistics Norway, 2022). Enrolling in childcare represents the first transition in a toddler’s life, and it is assumed that the experiences during this transition lay the foundation for children’s well-being in childcare and later transitions (Brooker, 2008; Dunlop, 2015). In the present study, we aim to explore toddlers’, parents’, and professional caregivers’ perceptions of the process of transition from home to childcare. There has been growing recognition that children’s transition between different contexts should be understood as a process rather than an event. A fundamental goal of the transition to childcare is to help children to be settled in childcare centers and develop a feeling of well-being and belonging (Broström, 2005). This study aimed to gain greater insight into the transition process that occurs when toddlers start in childcare.

In the bioecological model, Bronfenbrenner and Morris (2006) address development as the interrelatedness and interactions of an individual with the environment over time. They describe transition as an ecological concept where children’s microsystems (home and childcare center) are linked together in a network (the mesosystem) and influenced by the wider society—the macrosystem (e.g., length of paid parental leave and access to childcare centers). A transition from home to childcare implies a change in setting, relationships, routines, and roles, imposing demands on both children and families. However, according to an ecological approach, strong mesosystem links (e.g., different priming activities, such as visits to the childcare center, home visits, bringing a transactional object, and different kinds of parental involvement) between the home and childcare center can ease the transition process (Rimm-Kaufman and Pianta, 2000). To understand what happens in the microsystem, we also draw on the attachment theory, framing the importance of close relationships in child development (Bowlby, 1982; Sroufe, 2005; Ereky-Stevens et al., 2018).

Both home and childcare settings should be able to adapt and change in response to how well children adjust to childcare (Dunlop, 2015). Some children are more vulnerable to change, and transition can then cause frustration and worry, which may affect later emotional development, behavior, and learning capacity if they are not addressed and taken seriously (Kienig, 2002). However, starting childcare represents a positive life event for most children, and promotes new possibilities for change, development, learning, and joy (Brooker, 2008; Dunlop, 2013; Degotardi and Pearson, 2014). Transitions where children are given appropriate support and help can lead to the development of resilience and “transition skills” (Dunlop, 2015). Further, in positive transitions, children, families, and professional caregivers are given opportunities to be agents of change rather than subjects of transition routines outside their influence (Fabian and Dunlop, 2007). Building on this understanding, Urban et al. (2012) stated that we need competent childcare systems in which the continuity and progression of children’s emotional and social development, and learning are supported by transition-aware professionals.

Toddlerhood is a period characterized by significant changes across all developmental domains. Brain development is particularly rapid in the first 2 years (Hart, 2018). Because of their immature brain, toddlers need a lot of support to regulate physical states, emotions, behavior, and cognitive functions (Schore, 2015). Toddlers’ self-regulatory skills are developed in mutually regulated interactions within secure adult-child relationships, for example, when they are overwhelmed by difficult feelings or going to explore an unknown environment (Sroufe, 2005; Schore and Schore, 2008). Toddlers develop secure attachment towards adults (both parents and professional caregivers) that are sensitive and responsive to their basic needs, particularly their feelings (Bowlby, 1982; Powell et al., 2014; Ereky-Stevens et al., 2018). In secure attachment relationships, toddlers receive comfort and support when needed, and their emotional well-being is promoted. The key worker approach in childcare centers is based on attachment theory and includes a system in which one or two professional caregivers have special responsibility for a small group of children (Brooker, 2008). This system may promote toddlers’ well-being in childcare, in addition to positive collaboration between parents and professional caregivers, and stability in the teacher group (Ebbeck and Yim, 2009; Ebbeck et al., 2015).

Most toddlers are curious and active when facing something new. They have an internal drive to explore and learn, and a new environment may encourage positive feelings and new possibilities for young children (Degotardi and Pearson, 2014). However, to be able to branch out to explore the new childcare settings, toddlers need to have a secure base to return to when they need help or reassurance (Powell et al., 2014; O’Connor, 2017). Parents who are with their child during priming activities and when they start in childcare can function as a secure base, supporting their toddler’s transition process and the development of a relationship with their professional caregiver (Ahnert et al., 2004). This process can be described as establishing a “caring triangle” between the child, parent, and caregiver (Brooker, 2008; Brooker, 2010). When parents are no longer present in the childcare center, toddlers need their key person or another professional caregiver to help regulate their feelings and act as a secure base that supports their further exploration of new toys, materials, and relationships (Ebbeck et al., 2015). Many toddlers seem to be very interested in new toys when they start childcare (Thyssen, 2000), and shared attention towards these toys may ease professional caregivers’ process of becoming familiar with the child.

Cortisol is a product of the hypothalamic–pituitary–adrenal axis (HPA), and central to our stress response system. Cortisol has effects on both body and brain function (Gunnar and Cheatham, 2003), and the HPA axis is described as having a major role in how stress “gets under the skin” (Engel and Gunnar, 2020). The amygdala is found to be activated during and after elevations of cortisol, indicating that cortisol is related to learning, emotions, and memory (Harrewijn et al., 2020). Cortisol levels change in a diurnal cycle, with the highest values in the morning and a steady decline during the day (Gunnar and Herrera, 2013). This pattern has also been observed in young children (Watamura et al., 2004; Wesarg et al., 2022). During the day, cortisol levels rise when a situation is perceived as overly demanding. Rising cortisol levels are recognized as an indicator of social stress (Kirschbaum and Hellhammer, 1999; Gunnar and Quevedo, 2007). Assessment of toddlers’ cortisol levels during the transition to childcare may therefore be a suitable method to evaluate their experience during this phase. In childcare, cortisol levels are found to have an atypical development, in that they are somewhat higher in the afternoon as compared to the morning. This primarily applies to toddlers (Vermeer and van Ijzendoorn, 2006; Sumner et al., 2010; Tervahartiala et al., 2021), and is also found in Norwegian childcare centers (Drugli et al., 2018; Nystad et al., 2022).

To our knowledge, only four studies have investigated toddlers’ cortisol levels during their transition to childcare (Ahnert et al., 2004; Bernard et al., 2015; Nystad et al., 2021; Ahnert et al., 2022). Ahnert et al. (2004) found that when mothers were present and the child was securely attached to the mother, cortisol levels in childcare only showed a slight elevation compared to those at home. However, in the first period, separated from their mothers, all toddlers in their sample showed relatively high cortisol levels. In another study, Bernard et al. (2015) found rising cortisol levels in the afternoon during the first 10 weeks of childcare. Children aged 18–60 months showed higher levels than infants and elementary school children did. In a recent study (Nystad et al., 2021), toddlers showed elevated cortisol levels in the afternoon during the first weeks of childcare, with a peak on the second day without parents present and lower levels four to 6 weeks after enrollment. In the evening, when at home, the cortisol levels were low throughout the transition period. Toddlers younger than 14 months of age showed somewhat higher cortisol levels than older ones. In another recent study by Ahnert et al. (2022), cortisol levels were measured four times during the day for the first, second and fourth month after starting childcare. Many toddlers showed what was defined as a stress profile in the change in cortisol levels during the day in the first phase of the transition period. After 4 months, very few children showed this stress profile, probably because they adapted positively to the childcare setting.

There is little evidence on the association of higher cortisol levels with delayed functioning and development in children attending childcare, and more research is needed (Vermeer and Groeneveld, 2017; Tervahartiala et al., 2021). However, one Norwegian study revealed an association between toddlers’ well-being and change in cortisol levels during a year in childcare (Nystad et al., 2022). Toddlers with lower well-being scores at four to 6 weeks after starting in childcare, showed higher morning and afternoon values of cortisol during the year as compared to toddlers with higher well-being scores. The latter also showed higher cortisol levels at home in the evening, particularly at the end of the childcare year. Further, atypical diurnal cortisol patterns in early childhood seems to precede later internalizing problems (Saridjan et al., 2014).

Some qualitative studies have investigated the perspectives of parents and professional caregivers regarding toddlers’ adaptation during the transition period. In an early study, Dalli (1999) explored how five mothers perceived their toddlers’ process when they began childcare. The mothers described in interviews and journals that their toddlers needed them as a source of security as they became familiar with the routines and people in childcare. Further, they described their role as a bridge between the known and unknown for their children, but they also underlined the importance of standing back, such that the toddlers could explore new relationships in the childcare center. According to these mothers’ view, the toddlers could be said to have adapted to childcare when they no longer cried when dropped at the center in the morning or when they were able to be comforted by their professional caregivers when they cried during daily separations.

Qualitative interviews of mothers in a previous mixed-method study revealed that they described most of the toddlers as showing an easy transition to childcare (Swartz et al., 2016). Interestingly, some mothers described a more difficult transition for themselves than for their children Easy transitions were described by the mothers in this study as being associated with the young age of the child, professional caregivers that offered active support to the child during the transition, and routines (sleep and meals) in the childcare centers that matched the child’s routines at home. Difficult transitions were found to be associated with social fearfulness in the toddlers, maternal depressive symptoms, and maternal distress reactions to children’s distress in the quantitative part of the study.

In a small qualitative study, Brooker (2008) described how professional caregivers perceived three different toddlers adapting differently to a childcare center in London. One child was described as having a very easy transition, while the other two struggled during their first week of childcare. However, after four to 6 weeks these three toddlers were described as fully adapted to childcare in that they had developed positive relationships with their key worker and the rest of the staff; in addition to at least some of their peers, they knew the routines in childcare and showed positive feelings both when they entered childcare in the morning and during the day.

In a more recent study, Schärer (2018) explored four Canadian professional caregivers’ perspectives on toddlers’ transitions to childcare. She found that these teachers did not want to get too close to the children because they did not want them to be “over-attached.” They wanted to balance the needs of an individual child with the needs of the whole group.

Existing knowledge on toddlers’ transition to childcare is incomplete and further study is required on the perspectives and factors that support a “toddler friendly” transition (Brooker, 2008; O’Connor, 2017). For example, more data is required about when cortisol levels peak during the transition phase and how experiences during the childcare day may have spill-over effects to the home environment.

Because the transition to childcare involves multiple participants, the present study explores changes in cortisol levels in the toddler group (toddlers’ perspective) and the perspectives of parents and professional caregivers during the transition period. By using a mixed method approach, including both quantitative and qualitative data from different informants, we aimed to get a better understanding of toddlers’ transition to childcare across a 3 months’ period. Our research questions were: (1) What are the changes in toddlers’ levels of cortisol during the first months in childcare? (2) How do professional caregivers and parents perceive toddlers’ transition process during the first months in childcare?

## 2. Materials and methods

### 2.1. Design

Mixed methods are based on a pragmatic perspective (Creswell and Clark, 2011) that combines both quantitative and qualitative data in the same study, making it possible to draw on the strengths of both approaches.

In this study, we used a convergent mixed-method design (Creswell and Clark, 2011), also referred to as a convergent parallel design because all data is collected during one phase (Meixner and Hathcoat, 2019). The strength of this design is its potential to offer a more comprehensive understanding of a phenomenon. In the present study, quantitative (toddlers’ cortisol levels) and qualitative (notes from parents and key workers) data were collected separately on the same 6 days across the transition period and were prioritized equally. Quantitative and qualitative data were analyzed separately and independent of each other. In the Discussion section, we compare the two sets of results and interpret the extent to which they together create a better understanding of toddlers’ transition to childcare.

### 2.2. Procedure and participants

The present study is part of a larger project, “Little and new in childcare” (2018–2023), focusing on the development of an appropriate model for toddlers’ transition to childcare in the municipality of Trondheim, Norway. The model comprises four to eight scheduled visits to the childcare center before the start (1 h once a week during the spring), and the accompaniment of one parent for at least the first 5 days of childcare. In the first weeks without parents, the parents were encouraged to pick up their toddlers early from childcare. In addition, the keyworker approach (O’Connor, 2017) has been implemented in the childcare centers. In most centers, two key workers are responsible for three to four children during the transition, focusing on establishing a relationship with these children and their parents.

Data in the present study were collected in autumn 2021, and 32 childcare centers with 46 toddler classrooms participated voluntarily. Parents and professional caregivers of 120 toddlers who started in these 32 childcare centers in August and September 2021 were invited to provide written consent for saliva sampling and writing of notes during the transition phase. One to five toddlers were invited from each classroom because of the heavy workload for professional caregivers after the COVID-19 pandemic. If the parents of any child did not provide consent, parents of another in the same childcare department were approached.

Of the 120 toddlers enrolled in the study, seven were excluded because their saliva samples could not be collected, either because the child started in another childcare center, or would not cooperate for sample collection. Our final sample consisted of 113 toddlers (53.5% girls) in 46 toddler groups across 32 different childcare centers. Mean child age when starting childcare was 14.6 months (SD = 2.49); most children (87.4%) had a Norwegian language background, and 64% had a sibling. The average number of children in the childcare groups was 14.5 (SD = 2.47), and new toddlers entering these groups in autumn 2021 was 7.24 (SD = 3.01). In Norway, the child to caregiver ratio in toddler classrooms is regulated by the authorities and fixed at 3:1. At least one note each was written by 101 professional caregivers (96% keyworkers; 53.4% with a bachelor’s degree) and parents (64% mothers) of 87 children during the transition phase.

Both qualitative and quantitative data were collected on day two in the childcare center (1), day five in the childcare center (2), day two without a parent present (3), day nine without a parent present (4), day 12 without a parent present (5), and at a 3-month follow-up (6).

### 2.3. Saliva samples

Sampling kits with test tubes, swabs, and written instructions were delivered to the childcare centers by the project coordinator. Salimetrics’ SalivaBio Children’s Swab was used, which is intended for saliva sampling and has been validated for the analysis of cortisol (Salimetrics, 2019). Saliva samples were collected by the professional caregivers at 10 a.m. and 3 p.m. at the childcare centers. Sampling was performed in a playful manner to ease the toddlers through the process. Stimulants were not used. The samples were stored at the childcare center in a household freezer until they were collected by a research assistant. The cortisol laboratory at the University of Trier, Germany, analyzed the saliva samples for cortisol levels using a competitive solid phase time-resolved fluorescence immunoassay with fluorometric end-point detection (DELFIA; Dressendörfer et al., 1992). The samples were destroyed after analysis.

We received 1,000 samples from the laboratory, constituting 73.7% of 1356 possible samples. Seventy nine (5.82%) samples had been excluded by the laboratory because they contained to little saliva for analysis. Furthermore, 67 (6.7%) samples were excluded before the statistical analysis because of illogically high cortisol values. We excluded cortisol values > 30 nmol/l on the recommendations of the laboratory in Trier and the one at St. Olav’s Hospital in Trondheim. High cortisol levels may reflect sickness, medication, or contamination. In total, 933 (68.8%) cortisol values from 113 children were available for analysis. Seventeen (15%) children had a complete set of 12 cortisol values, whereas the rest (*n* = 96) were missing one or more values.

### 2.4. Notes from parents and professional caregivers

**Figure 1:**
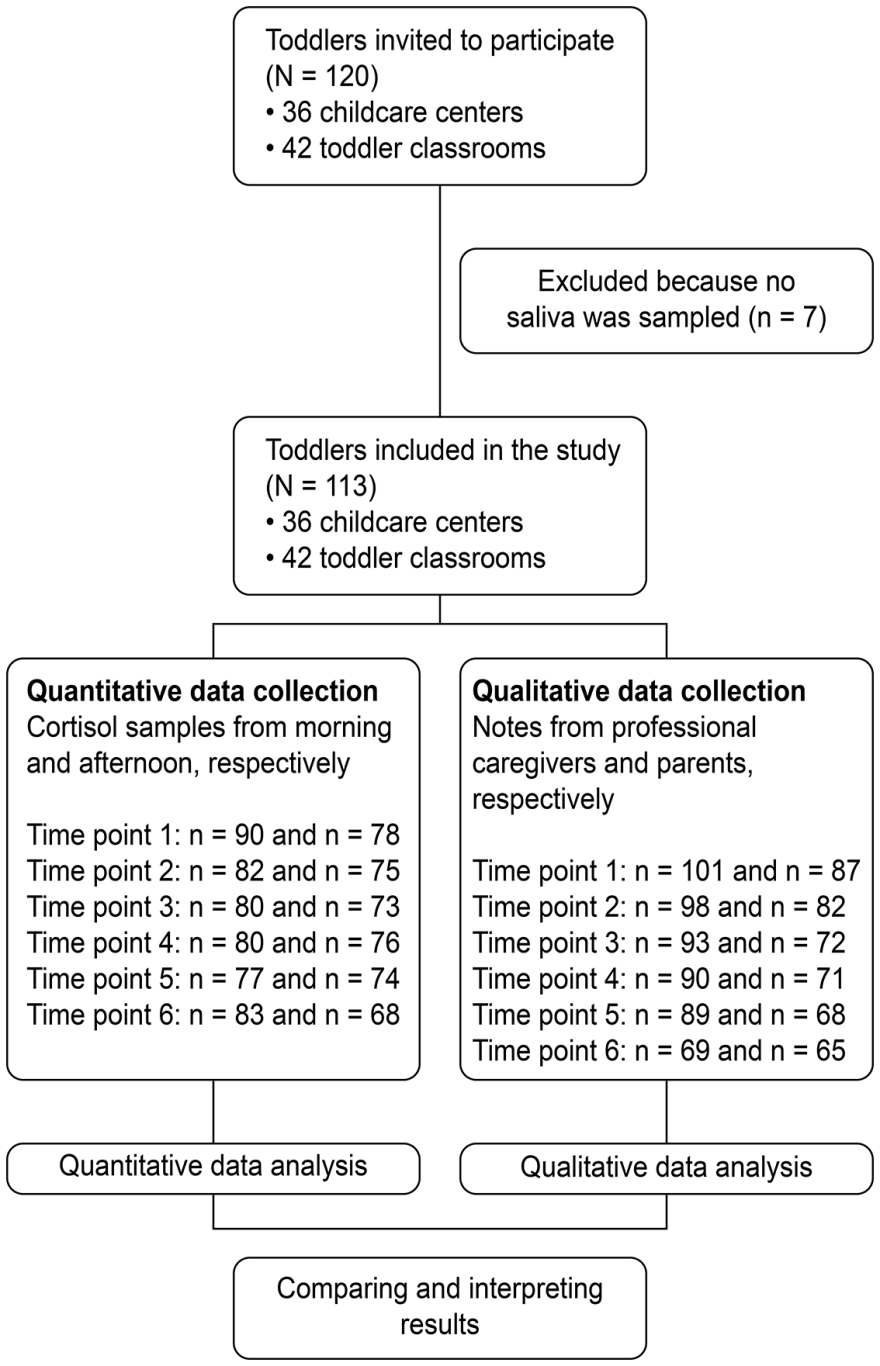
Flow-chart.

Professional caregivers and parents were asked a few open-ended questions about the transition process on the same days as the saliva samples were collected. Our questions were derived from existing theory and research on transition to childcare: what the child, parents, and professional caregivers had done in childcare (the first week), how separation and picking-up worked out (from week two), how well the professional caregivers perceived themselves as understanding the toddlers’ needs and signals (all days), perceptions of children’s well-being in childcare (all days), and how toddlers functioned at home after a day of childcare (from week two). Most notes were from half a page to one page in length per day. During the first 4 weeks, the response rate was relatively high, whereas it was quite low at the 3-month follow-up. See flowchart, Figure 1.

### 2.5. Ethics statement

Participation of the childcare centers, professional caregivers, and parents was voluntary. Professional caregivers and parents provided written consent, and parents provided written consent for their child’s participation as well. Consent could be withdrawn at all stages of the study. Professional caregivers were instructed not to collect the saliva samples if the child did not want to participate in the sampling procedure.

This study was approved by the Regional Committee for Medical and Health Research Ethics in Middle Norway, Norwegian University of Science and Technology (NTNU), Trondheim, Norway.

### 2.6. Data analysis

#### 2.6.1. Quantitative analysis

Our quantitative data were analyzed using a linear mixed model with the base 10 logarithm of cortisol concentration in nmol/l as the dependent variable and the individual as the random effect. The timepoints, or days post enrolling in childcare that measurements were taken, were entered as a six-category covariate day-2 in the childcare center (1), day five in the childcare center (2), day two without parent present (3), day nine without parent present (4), day 12 without parent present (5), and three-month follow-up (6)), and time of day [morning (10 am) and afternoon (3 pm)], and the interaction effects of day and time of day were included as fixed factors. Because cortisol levels are found to vary between age-groups (Watamura et al., 2004; Vermeer and Groeneveld, 2017; Nystad et al., 2021), analysis was controlled for the children’s age.

We did not impute missing variables because the linear mixed model included every data point of cortisol in the analysis, regardless of the number of missing data points from the same individual (Krueger and Tian, 2004). The normality of the residuals was checked by visual inspection of the QQ plots. We regarded two-sided *p*-values < 0.05 as statistically significant and reported 95% confidence intervals (CI) when relevant. Analyses were performed using IBM SPSS Statistics for Windows, Version 28.0 (IBM Corp., Armonk, NY, United States).

#### 2.6.2. Qualitative analysis

Thematic analysis was used to analyze the qualitative data (Braun and Clarke, 2012). The goal of our qualitative data analyses was to explore how parents and professional caregivers perceived the transition process. Each of the 6 days selected from the transition phase were analyzed separately.

The first and fourth authors conducted the thematic analysis in six steps as described by Braun and Clarke (2006, 2012) and Maguire and Delahunt (2017):

1. *Familiarization* refers to getting well-acquainted with the data. This was an important phase in the present study because of the large number of notes. A lot of time was spent reading and rereading the textual data. Notes about specific data items as well as the entire dataset were made during the reading process and discussed in several meetings between the two coders.
2. *Generating initial codes.* This step involved making sense of the data by systematically identifying their meaning throughout the dataset. In this step, the coders worked separately with the texts from parents and professional caregivers. They looked for codes that told them how the children were doing both in childcare and at home during the separation phase. In this way, the coders had a theoretical, and somewhat deductive, orientation to coding but concrete predefined codes were not used. The codes came from the data and the orientation towards coding, therefore, was also somewhat inductive. Further, the codes were semantic, and they captured the meaning in a language close to the participants’. All codes from both coders were used in this step, resulting in 74 different codes. Although some codes were quite similar, they were not discarded in this phase.
3. *Constructing potential themes.* In this step, potential themes were created with the intention of telling an insightful story about the data and our research question. Codes from both parents and professional caregivers with similar meanings and overlapping content were clustered into themes for each of the transition days. The coders tried to look for themes that together told a story about the transition process. The coders first worked independently to find potential themes, then they met and discussed the themes. The coders adopted a pragmatic orientation towards intercoder agreement, however, the agreement between the two coders was found to be high. Disagreements were mainly regarding the names of the potential themes, not the content. The two coders then discussed the potential themes and agreed on how to modify them. Assorted colors and visual maps were used to get an overview of the codes and visualize their grouping into meaningful themes. The potential themes were predominantly descriptive, in that they indicated the patterns in the data. Several themes were accepted in this step, such as “*Calm day,” “Parents take care of the child,” “Keyworker observes,” “Not all parents present,” “Key worker more responsive,” “Tired at home.”*
4. *Refining and defining themes.* This step involved reviewing the different codes and themes in addition to the text extracts connected to each code and theme. In this step, the coders worked collaboratively with the data from both parents and professional caregivers. The coders discussed if the themes made sense, if the data supported the themes, and if themes overlapped. Potential themes for each transition day were collapsed into two to three themes per day.
5. *Revising and naming themes*. In this step the coders worked collaboratively on the themes from step four, trying to find one or two overarching themes for each of the six transition days. The coders tried to name the themes in a manner that showed the essence of each transition day. For most days, one theme indicating the process in childcare and one theme describing the children’s functioning at home were used. The revised and overarching themes were: (1) “*Parents as secure base,”* (2) “*Change in roles between parents and keyworkers*,” *“Tired at home,”* (3) “*A bit unfamiliar,” “Tired at home”* (4) “*Adaptation in progress – but some struggle,” “Tired at home”* (5) “*Well-being for most children,” “Tired at home,”* and (6) *“Adapted in childcare,” “Still a bit tired at home.”*
6. Writing up the thematic analysis.

## 3. Results

In this section the quantitative and qualitative results are presented separately.

### 3.1. Results of the quantitative linear mixed model analysis

**Figure 2:**
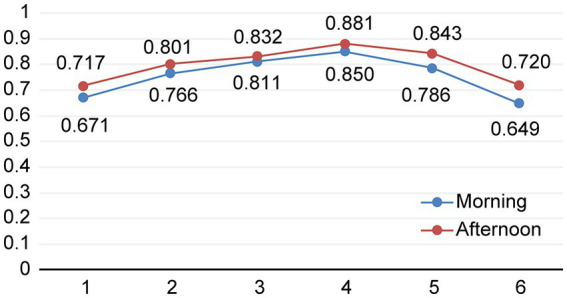
Change in estimated marginal means of cortisol in nmol/l (log 10-transformed) during the transition period. 1 = second day in childcare (accompanied by parents), 2 = day 5 in childcare, 3 = day 2 separated from parents, 4 = day 9 separated from parents, 5 = day 12 separated from parents, 6 = after 3 months.

Statistically significant main effects were found for timepoint (*p* < 0.001) and time of day (*p* = 0.015). No interaction was found between timepoint and time of day (*p* = 0.974). Table 1 shows the estimated marginal means of log 10-transformed cortisol values (nmol/l) for each timepoint and time of day, and Figure 2 shows the changes in cortisol levels across the days and times of the day.

**Table 1:** Estimated marginal means of log 10-transformed cortisol values (nmol/l).

Day	Time	*n*	Mean	95% confidence interval
1 (second day in childcare)	Morning	90	0.671	0.609–0.651
	Afternoon	78	0.717	0.651–0.783
2 (day 5 in childcare)	Morning	82	0.766	0.702–0.830
	Afternoon	75	0.801	0.734–0.869
3 (day 2 without parents in childcare)	Morning	77	0.811	0.744–0.878
	Afternoon	73	0.832	0.764–0.900
4 (day 9 without parents in childcare)	Morning	80	0.850	0.785–9.15
	Afternoon	76	0.881	0.815–0.948
5 (day 12 without parents in childcare)	Morning	77	0.786	0.720–0.851
	Afternoon	74	0.843	0.775–0.911
6 (after 3 months)	Morning	83	0.649	0.609–0.713
	Afternoon	68	0.720	0.651–0.783

#### 3.1.1. Changes throughout the days

In the morning, an increase in cortisol levels from timepoint 1 and timepoint 2, 3, 4 and 5 were found (*p*-values were 0.023, 0.001, < 001, and 0.007, respectively). No difference was found between timepoint one and six.

In the afternoons, there was an increase in cortisol levels from timepoint 1 to timepoint 3 (*p* = 0.010), 4 (*p* < 0.001) and 5 (*p* = 0.005). Cortisol values at timepoint 2 and 6 did not differ from timepoint 1.

#### 3.1.2. Changes throughout time of day

There were no statistically significant differences between the morning and afternoon cortisol levels on any of the six transition days.

### 3.2. Results of the qualitative thematic analysis

Qualitative analysis of the notes from the parents and professional caregivers showed gradual positive changes in the children’s emotions, behavior, and well-being during the first 4 weeks in childcare. A somewhat surprising finding was that many parents described the children as being very tired at home in the evenings, while tiredness was perceived to a smaller degree by caregivers in childcare. After 3 months most toddlers seemed to have adapted well to the childcare center.

Here, we describe themes that emerged from our analysis for each of the childcare days, the nuances within each theme as perceived by parents and professional caregivers, and quotes that illustrate the theme.

#### 3.2.1. Day two with parents in childcare

##### 3.2.1.1. “Parents as secure base”

Parents played an important role as a secure base for their children during this day, as perceived both by the parents themselves and the professional caregivers. Parents stayed close to their children during this day and most handled all routine situations, such as meals, changing diapers, and sleeping. Many toddlers were described as being busy exploring the childcare environment, particularly the toys, with their parents as a secure base. Other toddlers mostly stayed close to their parents, observing what was happening around them. Keyworkers were in the proximity of the parents and toddlers, and interacted with the toddlers when they found it appropriate. They observed interactions between parents and children to become familiarized with the child’s routines, needs, and signals. Day two with the parents present seemed to be an easy and calm day for the toddlers.

*He has mostly been busy with the toys, and I have been close by.* (Mother).

*The day has been calm, the father has taken care of his child and I have mostly been nearby observing.* (Keyworker).

*I was near the child and parent the entire day and offered the child some help.* (Keyworker).

With few exceptions, parents and professional caregivers described very few worries related to the toddlers’ start in childcare on this day.

#### 3.2.2. Day five with parents in childcare

##### 3.2.2.1. “Change in roles between parents and keyworkers”

On the fifth day in childcare, roles between parents and professional caregivers had changed when compared with day two. Keyworkers were now active toward the children, for example, they handled most or all the routine situations, played with the child, and offered comfort when needed. Parents were more withdrawn, and some parents left the department for shorter or longer periods of time.

I sat in a corner most of the day and the keyworker took care of the child. The child knew where to find me. He has been playing a lot. (Father).

*I left after breakfast and the keyworker took care of him. (Mother).*

I have taken care of her most of the day. We have developed positive contact and I feel I am able to comfort her. (Keyworker).

##### 3.2.2.2. “Tired at home”

A striking result on this day was that many parents described tired and quite exhausted children at home after the day in childcare. More than half the parents described their toddlers as being tired at home. Some toddlers were very frustrated and clingy, whereas others were in a good mood, even if they were described as being more tired than before starting childcare.

*She is very tired at home. She cries a lot, and I can’t leave the room.* (Mother).

*He is more tired in afternoons, but he is the same happy boy. (Father).*

#### 3.2.3. Day two without parents in childcare

##### 3.2.3.1. “A bit unfamiliar”

Results of the analysis revealed that most children seemed a bit unfamiliar in the new context on their second day without parents in childcare. Keyworkers described that they were making positive progress in becoming familiar with the new children, but they also felt that they did not always understand their signals and needs.

*It can be difficult to understand her signals. Sometimes tears are coming, but she doesn’t cry.* (Keyworker).

Keyworkers further described that the children’s well-being varied among the new toddlers.

*The child really seems to feel well. She is happy, seems secure and interacts with professional caregivers and peers.* (Keyworker).

*She needs more time to be secure, she needs us close by. She is not ready for play and exploration yet.* (Keyworkers).

Separation from their parents in the morning was described as easy for many children. For others, there was some crying when the parents left, but most children calmed down after a short period. A few children cried a lot and were very upset during separation and afterwards. Pick-up in the afternoon was described by most parents as a pleasant and easy situation. However, some described that the child started to cry when they arrived.

##### 3.2.3.2. “Tired at home”

The toddlers seemed to show more negative reactions at home than in childcare. At home, in the afternoon, more than half of the children were described as tired, hungry, clingy, or more frustrated than before starting childcare.

*He is tired and starts to cry often. He is also hungry and eats a lot.* (Father).

*Quieter than before, wants to be close to me, clingy.* (Mother).

*She is happy, but very tired.* (Mother).

#### 3.2.4. Day nine without parents in childcare

##### 3.2.4.1. “Adaptation in progress—but some struggle”

During the third week, many children were perceived to be more familiar with the childcare setting than the week before. In addition, professional caregivers’ descriptions indicated that they now understood the new toddlers quite well and were able to comfort most of them. However, they also described some children who struggled with adaptation to childcare.

Professional caregivers described high child well-being in general among the new toddlers, but also perceived low well-being in some individual children.

*It seems like he feels good here. He is happy, eats a lot and sleep well.* (Keyworker).

*She has had a positive development with regards to well-being. She has started to show her own will in some situations.* (Keyworker).

*She is distressed without her parents, and she get easily frustrated.* (Keyworker).

Children were generally described as having an easy separation from their parents in the morning. Others cried a bit but were easily comforted by their professional caregivers. A few children had a hard time when their parents left, particularly when they were not familiar with the professional caregiver present. Pick-up was described by parents as an easy and positive situation for most toddlers, but some still started to cry when their parents arrived at childcare.

##### 3.2.4.2. “Tired at home”

At home in the afternoon and evening, more than half of the toddlers still seemed to be quite exhausted while others were in a good mood.

*He wants to sit on our laps all afternoon, he seems very tired. (Father).*

*She has been happy and lively as usual.* (Mother).

#### 3.2.5. Day 12 without parents in childcare

##### 3.2.5.1. “Well-being for most children”

For most toddlers, this was their fourth week of childcare, and more children seemed now to enjoy themselves there, though a few individual children still had not settled down. Professional caregivers described that they felt it was easier to understand the toddlers’ feelings and comfort them this week.

*We have learned to read him, and we now understand his different signals.* (Caregiver).

*The child is now easier to comfort. She sometimes needs to be close and then one of us carry her or take her on our lap.* (Keyworker).

*He wants to be carried most of the time, and he cries when I (the keyperson) leave the room.* (Keyperson).

##### 3.2.5.2. “Tired at home”

Half of the children were still described as being tired in the afternoon and evening, as perceived by the parents. The rest were described as active and happy after the day spent in childcare.

*She is tired and wants to sit in our laps. She does not want me to leave the room.* (Mother).

*He is tired, fuzzy and hungry.* (Father).

*He has been as usual. He wants to play or read books, and he eats and sleeps well.* (Mother).

#### 3.2.6. After 3 months

##### 3.2.6.1. “Adapted in childcare”

After 3 months, the toddlers seemed to have fully adapted to childcare. Professional caregivers described high well-being among all the children during the day.

*He seems secure and happy. He plays a lot with other children.* (Professional caregiver).

*She runs into the classroom with a big smile every morning. We feel we have a close relationship with her.* (Professional caregiver).

##### 3.2.6.2. “A bit tired at home”

At this point, fewer children were described as tired and exhausted at home. Those who were still tired in the afternoons seemed to have less serious reactions compared to the previous weeks in childcare.

*Sometimes he is tired, but most often he is happy and playful.* (Mother).

*A bit cranky and tired, but not as much as before.* (Father).

## 4. Discussion

In this mixed-method study, we explored toddlers’ transition to childcare by combining quantitative (child cortisol levels) and qualitative (notes from parents and professional caregivers) data collected during the first 4 weeks of childcare and at a three-month follow-up. Our main finding was that the changes in the children’s cortisol levels and parents’ and caregivers’ perceptions of the transition process fit together well; they complement and expand each other in a meaningful way, and add to the knowledge regarding what happens during toddlers’ transition to childcare. Both our quantitative and qualitative data showed an easy start in childcare when parents were present and played an active role in caring for their own children. Further, both cortisol data and the notes indicated that the first 2 weeks without parents in childcare were demanding. The third week without parents in childcare seemed to be a turning point. In this week, cortisol levels decreased slightly, and professional caregivers described clear progress in the children’s well-being in childcare, with the exception of some individuals. On the other hand, parents perceived that many children were exhausted and tired at home in the evenings throughout the first 4 weeks. At the 3-month follow-up, cortisol levels returned to the same level as on the second day of childcare, and both parents and professional caregivers perceived that the toddlers had adapted positively to the childcare setting. Some children were still tired at home in the evenings, but to a lesser extent.

### 4.1. An easy start

Our finding of low levels of cortisol when toddlers were together with their parents in childcare corresponds with the findings of previous studies (Ahnert et al., 2004; Nystad et al., 2021). Notes from parents and professional caregivers showed that on day two of childcare, when all parents were with their child, the whole day was calm and pleasant. The toddlers themselves could choose if they wanted to stay close to their parents and observe or if they wanted to explore the childcare environment. The keyworkers stayed close to “their” toddlers and parents and used natural opportunities to try and establish a caring triangle comprising them, the child, and the parent (Brooker, 2008). Unlike the professional caregivers in Schärer’s (2018) study, the teachers in our study seemed to want to establish close and warm relationships with the toddlers, and often referred to concepts from attachment theory in their notes (Powell et al., 2014).

Another possible explanation for the low cortisol levels on the second day of childcare may be that most toddlers and parents in the present sample visited the childcare center several times before the first day. They had met some or all professional caregivers, other children and parents, and had played with toys and objects in their childcare department. In addition, a particular song had been used as a transition object between home and the childcare centers (Winnicott, 1953). Notes from parents and professional caregivers showed that many toddlers seemed to recognize the surroundings when they arrived at the childcare center in the autumn, indicating that different kinds of priming activities may be helpful for children to ease into the start of childcare (Fabian and Dunlop, 2007; Degotardi and Pearson, 2014; O’Connor, 2017). This may particularly be the case for toddlers, who are still developing in verbal communication and, therefore, need to experience what it feels like to be in the childcare center.

Cortisol levels in the morning were higher on day 5 in childcare than on day 2. This finding may be explained by notes showing that some parents left the department or the childcare center for a shorter or longer duration on day 4 and 5. Separation from parents has been found to be associated with elevated cortisol levels in toddlers (Ahnert et al., 2004; Nystad et al., 2021) and on day 5, some toddlers seemed to prepare themselves for such a separation when they entered childcare in the morning.

### 4.2. Separation, and gradually belonging

Toddlers’ cortisol levels showed rising values during the first 2 weeks without parents being present in childcare (see Figure 2). Our findings add to those of Ahnert et al. (2004) and Nystad et al. (2021), in which a peak in cortisol values was found in the first week, when toddlers were separated from parents. In the present study, we also measured the levels of cortisol the week after and found them to be even higher. Cortisol values started to decrease the following 2 weeks, indicating that the toddlers gradually adapted to their new context.

Keyworkers’ and parents’ notes shed light on the present findings of changes in cortisol values (Bryman, 2006). During the first 3 weeks of the separation phase (when parents no longer accompanied their child), keyworkers’ notes clearly showed a process in which they gradually became more and more familiar with individual toddlers’ signals, feelings, behavior, and needs. To establish a close relationship with a new child in childcare, time and repeated experiences of positive caring interactions are required (Degotardi and Pearson, 2014). Toddlers particularly need their emotional signals understood and met in appropriate ways (Gottman, 2011), and professional caregivers’ notes showed that it took time to understand all the new children.

The third week without parents in childcare seemed to be a turning point. In this week, cortisol levels decreased slightly, and professional caregivers described clear progress in the children’s well-being in childcare, with few exceptions.

### 4.3. Tiredness at home

Even if toddlers in the present sample seemed to have quite an easy start in childcare and gradually developed positive relationships and well-being, many parents described tired and clingy toddlers at home in the afternoons during the first month. Some toddlers were also very hungry, indicating that they had not eaten sufficient food in childcare.

These negative reactions at home can probably be explained by the fact that toddlers are easily overwhelmed by strong feelings and new experiences (Schore, 2015), and it can be difficult for them to be separated from their parents (Gunnar and Donzella, 2002). Young children may use a lot of energy in exploring the childcare setting, and because the professional caregivers still do not know all their signals and needs, they may not always receive the support they need. They may accumulate some negative feelings during the course of the day in childcare. Because cortisol levels have been found to decrease to low levels when toddlers arrive at home and relax after a day in childcare (Nystad et al., 2021), this may be one reason why they show more negative emotional reactions at home than during the day in the childcare setting. Clingy behavior towards parents and crying after a day in childcare may signalize a need for emotional regulation from those who know the toddlers best (Gottman, 2011). This may also signify that even though they had a good time in childcare, they still missed their parents.

### 4.4. Lower stress levels and higher well-being after 3 months

After 3 months, cortisol levels were again low, and both parents and professional caregivers perceived that most toddlers had adapted well to the childcare setting. In addition, previous studies have found that with passing time, stress levels in childcare are lowered, and children show more positive feelings in interactions with professional caregivers and peers (Datler et al., 2012; Nystad et al., 2021; Ahnert et al., 2022). In a recent study, Nystad et al. (2022) found that the development of stress levels throughout the childcare year were associated with professional caregivers’ perceptions of the children’s well-being during the first month of childcare and the child’s temperament, indicating that some individual children may continue to struggle with their adaptation to childcare in the longer run. Children’s well-being seems to be an indicator that needs to be considered seriously during the early period of childcare. Some children may show low well-being (Datler et al., 2012). Individual children who struggle with the transition process should receive early and competent support both from their professional caregivers and their parents.

According to the parents, most toddlers were no longer very tired in the afternoons after 3 months. This may be explained by the fact that cortisol levels in childcare were lower at this timepoint, and that toddlers had matured emotionally, socially, and cognitively from when they started childcare. However, parents reported that their toddlers showed negative reactions when the professional caregivers they were familiar with were absent, for example, because of sick leave.

Findings from the 3 month follow up need to be interpreted with some caution, because the amount of missing data were quite high at this time point.

### 4.5. Conclusion and implications for practice

Taken together, Our results indicate that we need to understand toddlers’ transition to childcare as a curvilinear process. It has an easy start with parents present, followed by a demanding phase, and after about 4 weeks, most toddlers seem to have settled in. An additional finding was that the children showed fatigue during the demanding phase, primarily at home.

This study provides some ideas for the development of toddler-friendly transition routines in childcare centers. Toddlers seem to benefit from priming activities, such as visiting the childcare center before their first day. Parents and professional caregivers should cooperate closely to establish a “caring triangle” between them and the child before parents leave the childcare center because toddlers need time before they can use professional caregivers as their secure base in childcare. During the first weeks without parents present in childcare, toddlers seem to need a lot of emotional support both at home and in childcare, and they should have shorter hours in the childcare center whenever possible. Both professional caregivers and parents should ensure that the toddlers experiences closeness and quiet activities at the end of the day in the childcare center and at home, respectively.

### 4.6. Strengths, limitations, and suggestions for future research

Few studies have focused on the transition process when young children start childcare, and our study adds to the existing knowledge in this field. An important strength of the present study is the use of a mixed-method approach. Quantitative and qualitative data complement each other and provide a more thorough understanding of toddlers’ transition to childcare. By using mixed methods, the findings can be more nuanced than by using only one type of data. Our quantitative data assesses empirical reality with precision, whereas the qualitative data provide more details (Li et al., 2000). Another strength is the relatively high number of toddlers who participated in the study.

The present study has several limitations. Our study did not include a control group of children who did not attend childcare. Therefore, we cannot be entirely sure that the observed changes in cortisol activity were produced by childcare. Many of the toddlers included in the study were of a young age and the stress reactivity of their HPA axis may not yet have been fully regulated. However, particularly the morning values seems to be very reliable also for young toddlers (Wesarg et al., 2022). Missing data (both quantitative and qualitative) may also have influenced our results. Furthermore, we were unable to control for food intake, waking times in the morning, nap times, and the use of corticosteroids, which could alter cortisol levels. We did not measure evening cortisol levels and did not know how much they decreased when the children arrived at home. However, in a previous Norwegian study, cortisol levels were found to be low in the evenings at home (Nystad et al., 2021). It is also a limitation that we did not have the resources to conduct qualitive interviews on each of the separation days. Interviews would probably have given more nuanced data as compared to notes. In the qualitative analysis, our subjective standpoints and preconceptions regarding toddlers’ transition to childcare may have affected our work. However, we were very careful regarding basing the findings on participants’ responses and not our own motivation throughout the analyzing process and interpretation of our findings. The fact that the quantitative and qualitative data fit well together, reduces the risk of bias in the qualitative analysis.

Further research on toddlers’ transitions to childcare is needed. For example, observations and interviews during the transition period could provide more nuanced knowledge about how different children adapt differently to the childcare setting and how parents and professional caregivers can offer appropriate and adequate support both at home and in childcare.

## Data availability statement

The datasets presented in this article are not readily available because qualitative notes are not digitized. Requests to access the datasets should be directed to may.b.drugli@ntnu.no.

## Ethics statement

The studies involving human participants were reviewed and approved by The Regional Committee for Medical and Health Research Ethics in Middle Norway, Norwegian University of Science and Technology (NTNU), Trondheim, Norway. Written informed consent to participate in this study was provided by the participants’ legal guardian/next of kin.

## Author contributions

MD, KN, and AB designed the study and collected the data. MD and SL analyzed the quantitative data. MD and AB analyzed the qualitative data. MD drafted the manuscript. KN, SL, and AB commented on the manuscript. All authors contributed to the article and approved the submitted version.

## Funding

The study was funded by Trondelag county authority, the municipality of Trondheim, and the Regional Centre for Child and Youth Mental Health and Child Welfare, Department of Mental Health, Faculty of Medicine and Health Science, Norwegian University of Science and Technology (NTNU).

## Conflict of interest

The authors declare that the research was conducted in the absence of any commercial or financial relationships that could be construed as a potential conflict of interest.

## Publisher’s note

All claims expressed in this article are solely those of the authors and do not necessarily represent those of their affiliated organizations, or those of the publisher, the editors and the reviewers. Any product that may be evaluated in this article, or claim that may be made by its manufacturer, is not guaranteed or endorsed by the publisher.
